# Estradiol mediates the interaction of LINC01541 and miR-429 to promote angiogenesis of G1/G2 endometrioid adenocarcinoma *in-vitro*: A pilot study

**DOI:** 10.3389/fonc.2022.951573

**Published:** 2022-08-05

**Authors:** Dan Qiao, Xiaoduo Qin, Haiyan Yang, Xuantong Liu, Libing Liu, Sufen Liu, Zhongzhi Jia

**Affiliations:** ^1^ Department of Gynecology, Dalian Medical University, Dalian, China; ^2^ Department of Gynecology, Changzhou No. 2 People’s Hospital, Changzhou, China; ^3^ Department of Interventional Radiology, Changzhou No. 2 People’s Hospital, Changzhou, China

**Keywords:** endometrioid adenocarcinoma, estradiol, LINC01541, angiogenesis, miR-200s

## Abstract

**Background:**

Endometrioid adenocarcinoma (EAC) is the most common subtype of endometrial cancer (EC) and is an estrogen-related cancer. In this study, we sought to investigate the expressions and mechanism of action of 17β-estradiol (E2) and long noncoding RNA (lncRNA) LINC01541 in G1/G2 EAC samples.

**Methods:**

The expressions of estrogen receptor β (ESR2), LINC01541, miR-200s, and VEGFA were evaluated using real-time PCR in human EAC tissues (n = 8) and adjacent normal tissues (n = 8). Two EC cell lines (Ishikawa and RL95-2) were selected for validation *in vitro*. Bioinformatics analyses and luciferase reporter analyses were performed to verify potential binding sites. qRT-PCR, Western blot, and CCK-8 were used to identify the regulatory mechanisms of related genes in cell biological behavior.

**Results:**

Compared with adjacent normal tissues, LINC01541 and miR-200s family (except miR-200c) were highly expressed in EAC tissues (n=8), while ESR2 and VEGFA were lowly expressed in EAC tissues (* *P* < 0.05; ** *P* < 0.01). *In vitro*: E2 inhibited the expression of LINC01541 and miR-429 in both cell lines, and estrogen antagonist (PHTPP) could reverse this effect, in addition, PHTPP could promote the proliferation of these two cancer cells, cell transfection LINC01541 also had this effect after overexpression of plasmid and miR-429 mimic. E2 promotes the expression of VEGFA in both cell lines, and PHTPP can also reverse this effect. LINC01541 interacts with miR-429 to promote the expression of each other, and both inhibit the synthesis of VEGFA in EAC cells after overexpression. Through the double validation of bioinformatics analysis and dual fluorescein reporter gene, it was confirmed that miR-429 targets the regulation of VEGFA expression (* *P* < 0.05; ** *P* < 0.01).

**Conclusion:**

E2 promotes the synthesis of VEGFA by altering the expression levels of LINC01541 and miR-429 in EAC, thereby affecting the angiogenesis process of EAC. Also, E2-mediated LINC01541/miR-429 expression may affect cell migration in EAC. In addition, we identified a reciprocal promotion between LINC01541 and miR-429.

## Introduction

Endometrial cancer (EC) is the sixth most common tumor type and the eleventh most common cause of death in women worldwide, with endometrioid adenocarcinoma (EAC) accounting for 80% of all EC cases and the majority of well and moderately differentiated endometrioid histology (G1/G2). The vast majority of EC cases are in postmenopausal women. Long-term estrogen therapy, premature menarche, delayed menopause, infertility, polycystic ovary syndrome, advanced age, obesity, hypertension, diabetes are all risk factors for EC (1, 2). It is well known that EC is an endocrine-related cancer and that estrogen plays a key role in the occurrence and progression of this disease. Two specific intracellular receptors, estrogen receptor α (ERα) (ESR1) and ERβ (ESR2), mediate the biological effects of estrogen, with 17β-estradiol (E2) acting in the body mainly through ESR2 (3, 4). Research has demonstrated that siRNA-mediated knockdown of ESR2 and treatment with the ESR2 antagonist PHTPP can induce proliferation of HEC-1A (EC cell line) and RL95-2 (EC cell line) cells (5).

Increasingly, studies have shown that long noncoding RNAs (lncRNAs) play a key role in many biological processes (6). One such lncRNA is LOC100505776, also known as LINC01541 (NR_038325.1). Mai and colleagues used the Ishikawa cell line as a tool cell to study endometriosis and found that E2 inhibited the expression of LINC01541 in Ishikawa cells (7). Along similar lines, miRNAs have been found to be dysregulated in many human cancers, acting as tumor promoters or suppressors (8). More specifically, the miR-200s (miR-200a, miR-200b, miR-200c, miR-141, and miR-429) are dysregulated in EC, affecting cell processes such as migration and invasion. Some research has found that the expression of miR-429 is up-regulated in EC tissues versus in adjacent tissues, and other studies have shown that silencing the expression of miR-429 significantly inhibits the growth of HEC-1A and Ishikawa cells (9, 10).

Angiogenesis also plays a vital role in tumor progression (11). Multiple studies have demonstrated that angiogenesis is regulated by vascular endothelial growth factor (VEGF), including VEGFA, VEGFB, VEGFC, VEGFD and VEGFE, which are part of the dimeric glycoprotein family. These factors are secreted by many types of cells, including cancer cells (12), and research has shown that the expression of VEGF mRNA in uterine tissues is significantly up-regulated after exposure to estradiol treatment (13). Studies have found that VEGFA is a potential target of miR-429 (14, 15). Taken together, we speculate that ESR2 affects the production of VEGFA by regulating the expression of LINC01541 and miR-429 in EAC tissue, and this pathway affects the occurrence and progression of EAC. In this study, we sought to explore the effect of E2 stimulation on Ishikawa and RL95-2 cells, as well as the effects of LINC01541 silencing and overexpression in both cell lines. Ishikawa and RL95-2 cells are estrogen-sensitive and represent a well differentiated EAC cell line and a moderately differentiated adenosquamous carcinoma cell line, respectively (16, 17). We also aimed to investigate the effects of LINC01541 on cell proliferation and VEGFA secretion.

## Materials and methods

### Patients and tissue collection

This study was approved by the Ethics Committee of our hospital. Patients provided informed consent for tissue sample collection before the study began.

From October 2020 to July 2021, both human EAC tissues and adjacent normal endometrial tissues were collected from 8 patients (mean age: 54.5 ± 5.0 y; tumor grade G1-G2) who underwent total hysterectomy recruited from the Changzhou No. 2 People’s Hospital Affiliated to Nanjing Medical University. The clinical information of all patients was shown in **Table 1**. Formalin-fixed paraffin embedded samples from each tumor were stained with hematoxylin-eosin and evaluated by two pathologists. Inclusion criteria were as follows: 1) patients had not received any hormone therapy in the previous 6 months, 2) patients were diagnosed as EAC (tumor grade G1-G2) according to the International Federation of Gynecology and Obstetrics (FIGO) surgical and pathological criteria. Exclusion criteria were as follows: The patient has other malignant tumors or gynecological diseases other than EC.

**Table 1:** Clinicopathological characteristics of the EAC patients included in the study.

Characteristic	All patients n=8	grade G1 n=4	grade G2 n=4
Mean age (y) (range)	54.5 (44-60)	52.5 (44-59)	56.5 (55-60)
BMI (kg/m^2^)			
<24.9 (normal)	2 (25.0%)	1 (25.0%)	1 (25.0%)
25.0-29.9 (overweight)	5 (62.5%)	2 (50.0%)	3 (75.0%)
>30.0 (obese)	1 (12.5%)	1 (25.0%)	_
Hypertension			
Yes	3 (37.5%)	1 (25.0%)	2 (50.0%)
No	5 (62.5%)	3 (75.0%)	2 (50.0%)
Diabetes			
Yes	2 (25.0%)	1 (25.0%)	1 (25.0%)
No	6 (75.0%)	3 (75.0%)	3 (75.0%)
FIGO stage			
IA	7 (87.5%)	4 (100%)	3 (75.0%)
IB	1 (12.5%)	_	1 (25.0%)
LVSI			
Yes	1 (12.5%)	_	1 (25.0%)
No	7 (87.5%)	4 (100%)	3 (75.0%)

LVSI, lymphovascular space invasion.

### Bioinformatics predictions

We used three bioinformatics software programs: RNAhybrid (https://bibiserv.cebitec.uni-bielefeld.de/rnahybrid), starBase v3.0 (https://starbase.sysu.edu.cn/index.php) and GEPIA (http://gepia.cancer-pku.cn/detail.php) to predict the expression of and associations between LINC01541, miR-200s, and VEGFA. The results of each program were screened, and common miRNAs were selected as candidate LINC01541-interacting miRNAs.

### Cell line culture and transfection

Ishikawa cells were obtained from Chuan Qiu Biotechnology (catalog number H044, Shanghai, China), RL95-2 cells were obtained from Zhong Qiao Xin Zhou Cell (ZQ0362, Shanghai, China). Neither cell line was contaminated. Ishikawa cells were treated with DMEM medium (C11995500BT, Gibco; Auckland, New Zealand) containing 10% fetal bovine serum (FBS) (10270-106, Gibco) and 1% penicillin/streptomycin (15140122, Gibco). RL95‐2 cells were cultured in DMEM/F12 medium (ZQ-604-100, Gibco) mixture supplemented with 10% FBS and 1% penicillin/streptomycin and 10 mg/ml insulin (CSP001-10, Zhong Qiao Xin Zhou Cell). Both cell lines were carried out at 37°C in 5% CO2 conditions. LINC01541 pCDNA 3.1 overexpression vector, LINC01541-specific siRNA, miR-429 mimic, and miR-429 inhibitor were synthesized by RiboBio (Guangzhou, China) (si-LINC01541: 5′-ATTCAATTGTTTTTAATCCAT-3′; si-NC: 5′-GGCTCTAGAAAAGCCTATGC-3′; has-miR-429 mimic: 5′-UAAUACUGUCUGGUAAAACCGU-3′; mimic negative control: 5′-UUUGUACUACACAAAAGUACUG-3′; has-miR-429 inhibitor: 5′-ACGGUUUUACCAGACAGUAUUA-3′; and inhibitor negative control: 5′-CAGUACUUUUGUGUAGUACAAA-3′).

### Hormone treatment

E2 (E8875, Sigma-Aldrich; St. Louis, USA) and PHTPP (ESR2 antagonist) (SML1355, Sigma-Aldrich) were dissolved in alcohol. The precultured Ishikawa and RL95-2 cells were then treated with various concentrations of E2 (0 [control], 10^-6^ mol/L, 10^-8^ mol/L, and 10^-10^ mol/L) and PHTPP (0 [control], 10^-3^ mol/L, 10^-4^ mol/L, and 10^-5^ mol/L). After hormone treatment, the cells were incubated with DMEM or DMEM/F12 medium for 24 h. The culture medium was then changed daily.

### Cell viability assay

The transfected cells were seeded into a 96-well plate at a density of 5×10^3^ cells per well. A total of 10 μL of cell counting kit-8 (CCK-8) reagent (CK04, Dojindo, Japan) was added to each well, and the cells were incubated for 1 h at 37°C and 5% CO2. The OD450 value for each well was detected using an ultramicro microplate spectrophotometer (BioTek Epoch; Santa Clara, CA, USA). Each group was assayed in triplicate at daily intervals after consecutive seeding for up to 72 or 96 h.

### Western blot assay

The Ishikawa and RL95-2 cells were homogenized in radio immunoprecipitation assay (RIPA) buffer (P0013B, Beyotime Biotechnology; Shanghai, China) using a standard method. Protein extracts (30 µg) were then transferred to polyvinylidene difluoride membranes (Millipore; Boston, MA, USA). The membranes were blocked and incubated with antibody for VEGFA (66828-1-Ig, Proteintech; Wuhan, China) (diluted at 1:1000) or Tubulin (200608, ZENBIO; Chengdu, China) (diluted at 1:5000) and then with horseradish peroxidase conjugated secondary antibody (511103, ZENBIO) (diluted at 1:5000). The membranes were developed using chemiluminescence substrate (A38555, Thermo Scientific; Shanghai, China) and exposed to X-ray films.

### Total RNA extraction and quantitative real-time polymerase chain reaction

Total RNA was extracted using Trizol reagent (T9108, Takara; Dalian, China), and first-strand cDNA was synthesized using HiScript II Q RT SuperMix for qPCR (+gDNA wiper) (R223, Vazyme; Nanning, China). The qRT-PCR was performed using SYBR-Green Premix (Q131-02/03, Vazyme) with specific PCR primers (RiboBio). The qRT-PCR for miRNAs was performed using miRNA Strand cDNA Synthesis Kit (MR101-02, Vazyme) and miRNA Universal SYBR qPCR Master Mix (MQ101-01/02, Vazyme), respectively. Two primers (h-LINC01541-90bp/h-LINC01541-120bp) were designed for LINC01541; qRT-PCR demonstrated that h-LINC01541-90bp was more efficient, and so this primer was used in subsequent experiments. GAPDH and U6 were used as internal controls. Fold changes were calculated using the 2^−ΔΔCt^ method. RT primer for ESR2, LINC01541 and VEGFA are premixed random primers. The remaining primer sequences are listed in the **Table 2** with the exception of the RiboBio miRNA and U6 primer (including RT primer) sequences, which are confidential to the company.

**Table 2:** Primers used in the study.

Primers of genes	Sequences (5'-3')
hESR2-81-F	TTCAAAGAGGGATGCTCACTTC
hESR2-81-R	CCTTCACACGACCAGACTCC
h-LINC01541-90bp-F1	GCTTCCTTGCCTTTTCTCCT
h-LINC01541-90bp-R1	TGAGGCTGGAAACAGAGGTT
h-LINC01541-120bp-F2	GAATGAAGCTTTGGGGTGAA
h-LINC01541-120bp-R2	CCCACAAATGCACATCTATCC
hVEGFA-91-F	TCGGGAACCAGATCTCTCAC
hVEGFA-91-R	TCTGTCGATGGTGATGGTGT
GAPDH-F	TGAAGGTCGGAGTCAACGGATTTGGT
GAPDH-R	CATGTGGGCCATGAGGTCCACCAC

### Luciferase assay

Ishikawa and RL95-2 cells were seeded into a 96-well plate at a density of 1.5×10^4^ cells per well. Subsequently, the cells were cotransfected with miR-429 mimic (5′-UAAUACUGUCUGGUAAAACCGU-3′)/negative control and wild-type/mutant VEGFA (pmirGLO-h-VEGFA-WT: 5′…CAGTATT…3′/pmirGLO-h-VEGFA-MUT: 5′…GTCATAA…3′) (RiboBio). After 48 h of transfection, a dual-luciferase reporter assay was performed according to the manufacturer’s instructions (E2920, Promega; Madison, WI, USA). Renilla luciferase activity was used for calibration.

### Rescue assay

To further study the relationship between LINC01541 and miR-429, we used Ishikawa and RL95-2 cells for rescue experiments. For the first set of experiments, we established 4 groups: a LINC01541 overexpression group, an miR-429 inhibitor group, a co-culture group (LINC01541 overexpression/miR-429 inhibitor), and a PBS group (control). For the second set of experiments, we then established another 4 groups: a knockdown of LINC01541 group, an miR-429 mimic group, a co-culture group (knockdown of LINC01541/miR-429 mimic), and another PBS group (control). Cells were then cultured in 24-well plates for 24 h for qRT-PCR, in 96-well plates for 24 h/48 h/72 h for CCK-8 analysis, and in 6-well plates for 48 h for Western blot analysis.

### Statistical analysis

In this study, GraphPad Prism 8.0.2 software was used to analyze data. All studies were verified by three independent experiments. Student’s *t*-test was used to analyze two sets of data, and one-way ANOVA was used to analyze multiple sets of data. Data were expressed as mean ± SD. *P* values less than 0.05 were used to represent statistical significance.

## Results

### Bioinformatics prediction and the expression of related genes in EAC tissues

**Figure 1:**
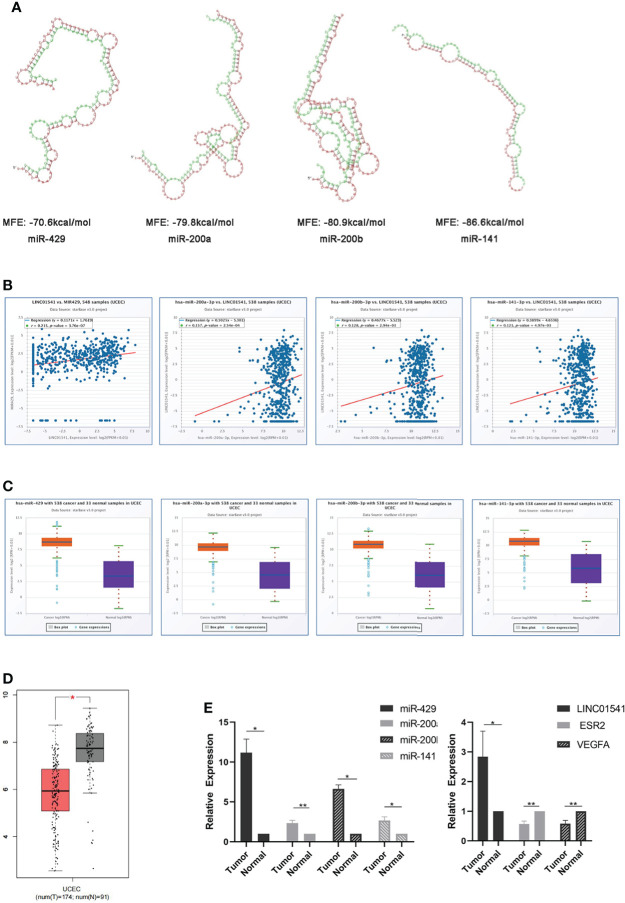
Bioinformatics prediction and the expression of related genes in EAC tissue specimens. **(A)** RNAhybrid software demonstrated that miR-429, miR-200a, miR-200b, and miR-141 can bind to LINC01541 (red: LINC01541; green: miRNAs). MFE = minimum free energy (the absolute value of which represents the binding degree of miRNA and target mRNA or lncRNA). **(B)** StarBase v3.0 demonstrated a positive correlation between miR-429 and LINC01541 in UCEC (*P*<0.01). Other members of the miR-200s family also demonstrated this trend. **(C)** starBase v3.0 demonstrated increased expression of miR-200s (except miR-200c) in UCEC. **(D)** Boxplot demonstrating VEGFA transcriptional expression in UCEC and normal uterus tissues from GEPIA. The red boxes represent UCEC (n = 174); the gray boxes represent normal tissue (n = 91). The y-axis indicates the log2-transformed gene expression level. **(E)** The expression levels of LINC01541 and miR-200s in G1/G2 EAC tissues (n = 8) were significantly higher than those in normal tissue (n = 8); however, both ESR2 and VEGFA were decreased in EAC tissue. (*P<0.05; **P<0.01).

RNAhybrid demonstrated that, with the exception of miR0200c, the miR-200s family was able to bind with LINC01541 (**Figure 1A**). The starBase v3.0 database also demonstrated this binding ability, as well as increased expression of miR-200s (except miR-200c) in uterine corpus endometrial carcinoma (UCEC) (**Figures 1B, C**). The expression of VEGFA was found to be down-regulated in UCEC compared with adjacent tissues (*P*<0.05) (**Figure 1D**). As shown in **Figure 1E**, LINC01541 was highly expressed in EAC tissues, suggesting that this could be a prognostic marker. The miR-200s family (with the exception of miR-200c) was also highly expressed in EAC tissues.

### E2 regulates the expression of LINC01541 and miR-429 and affects the progress of EAC

**Figure 2:**
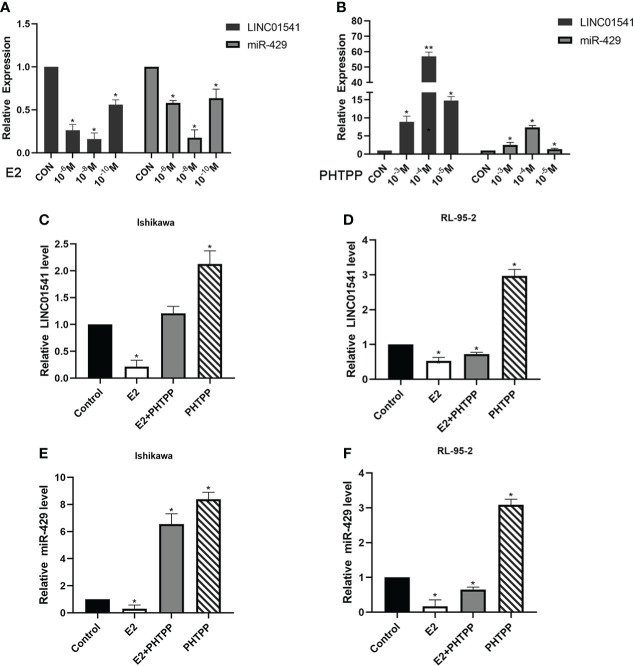
qRT-PCR was used to detect the effect of E2 and PHTPP in EAC cells. **(A)** The down-regulation of LINC01541 and miR-429 was most obvious when the concentration of E2 was 10^-8^ mol/L. **(B)** The down-regulation of LINC01541 and miR-429 was reversed most obviously when the concentration of PHTPP was 10^-4^ mol/L. **(C-F)** In Ishikawa and RL95-2 cell lines, E2 inhibited the expression of LINC01541 and miR-429, whereas PHTPP was able to reverse this effect. (*P<0.05; **P<0.01).

E2 (at an optimal concentration of 10^-8^ mol/L) was found to have an inhibitory effect on LINC01541 and miR-429, and PHTPP (at an optimal concentration of 10^-4^ mol/L) was found to reverse this effect in Ishikawa and RL95-2 cells (**Figures 2A–F**).

**Figure 3:**
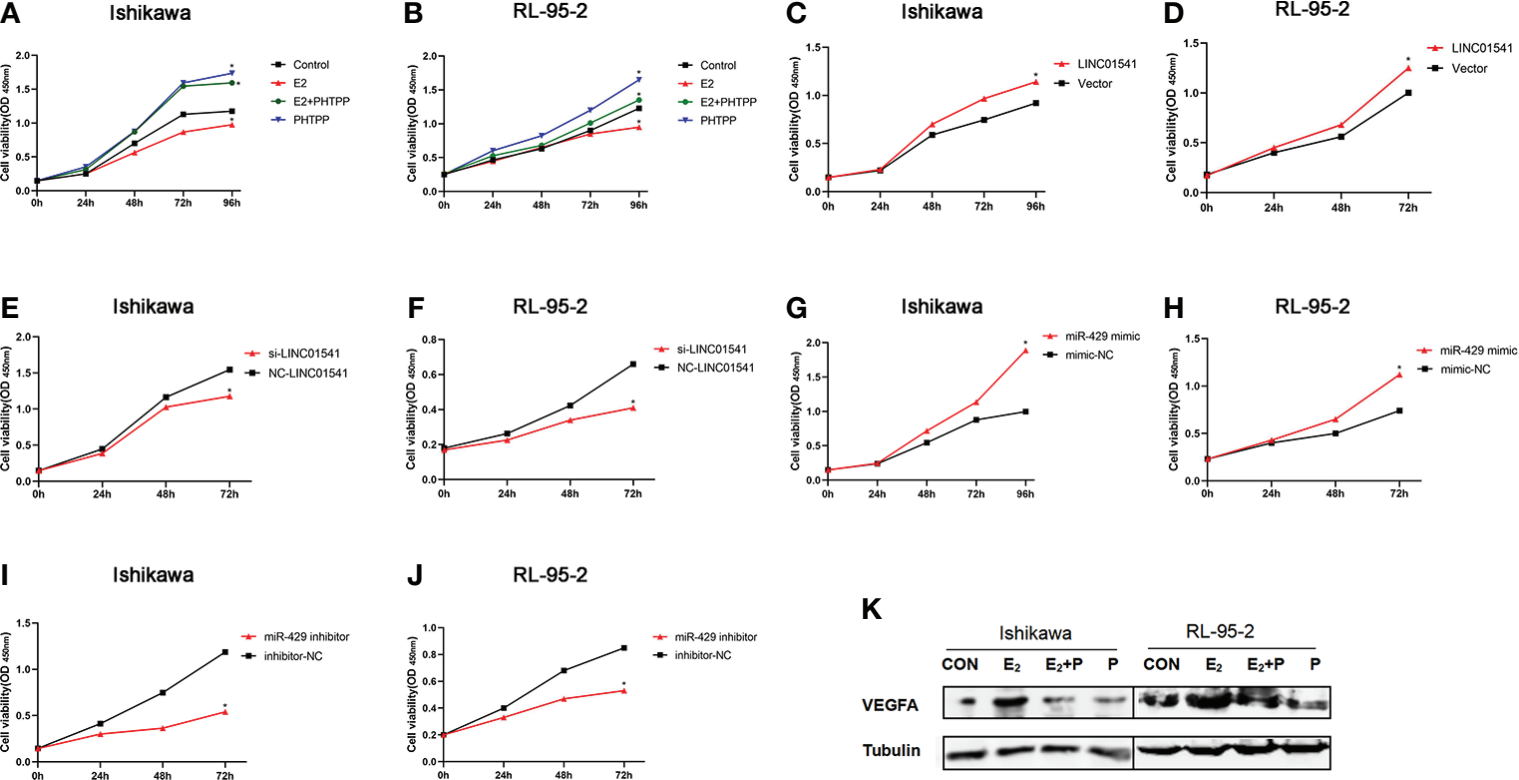
CCK-8 and Western blot analysis were used to analyze the effects of treatment. **(A, B)** In Ishikawa and RL95-2 cells, E2 inhibited cell proliferation, whereas PHTPP and a mixture of PHTPP and E2 promoted cell proliferation. **(C, D)** Overexpression of LINC01541 promoted proliferation of both cell lines. **(E, F)** Interfering with the expression of LINC01541 inhibited proliferation of both cell lines. **(G, H)** Overexpression of miR-429 promoted proliferation of both cell lines. **(I, J)** Silencing the expression of miR-429 inhibited proliferation of both cell lines. **(K)** Compared with the control group, E2 promoted the expression of VEGFA, whereas PHTPP inhibited the expression of VEGFA; a mixture of the two had an intermediate effect on VEGFA in Ishikawa and RL95-2 cells. (*P<0.05).

E2 slightly inhibited the proliferation of Ishikawa and RL95-2 cells, whereas PHTPP significantly promoted the proliferation of these cells (**Figures 3A, B**). As expected, both LINC01541 and miR-429 promoted the proliferation of EAC cells (**Figures 3C–J**), suggesting that the effect of E2 on the proliferation of EAC cells was not mediated by the regulation of LINC01541 and miR-429. Finally, E2 was found to promote the expression of VEGFA, with PHTPP able to reverse this effect (**Figure 3K**).

### The interaction between LINC01541 and miR-429 affects the expression of VEGFA in EAC

**Figure 4:**
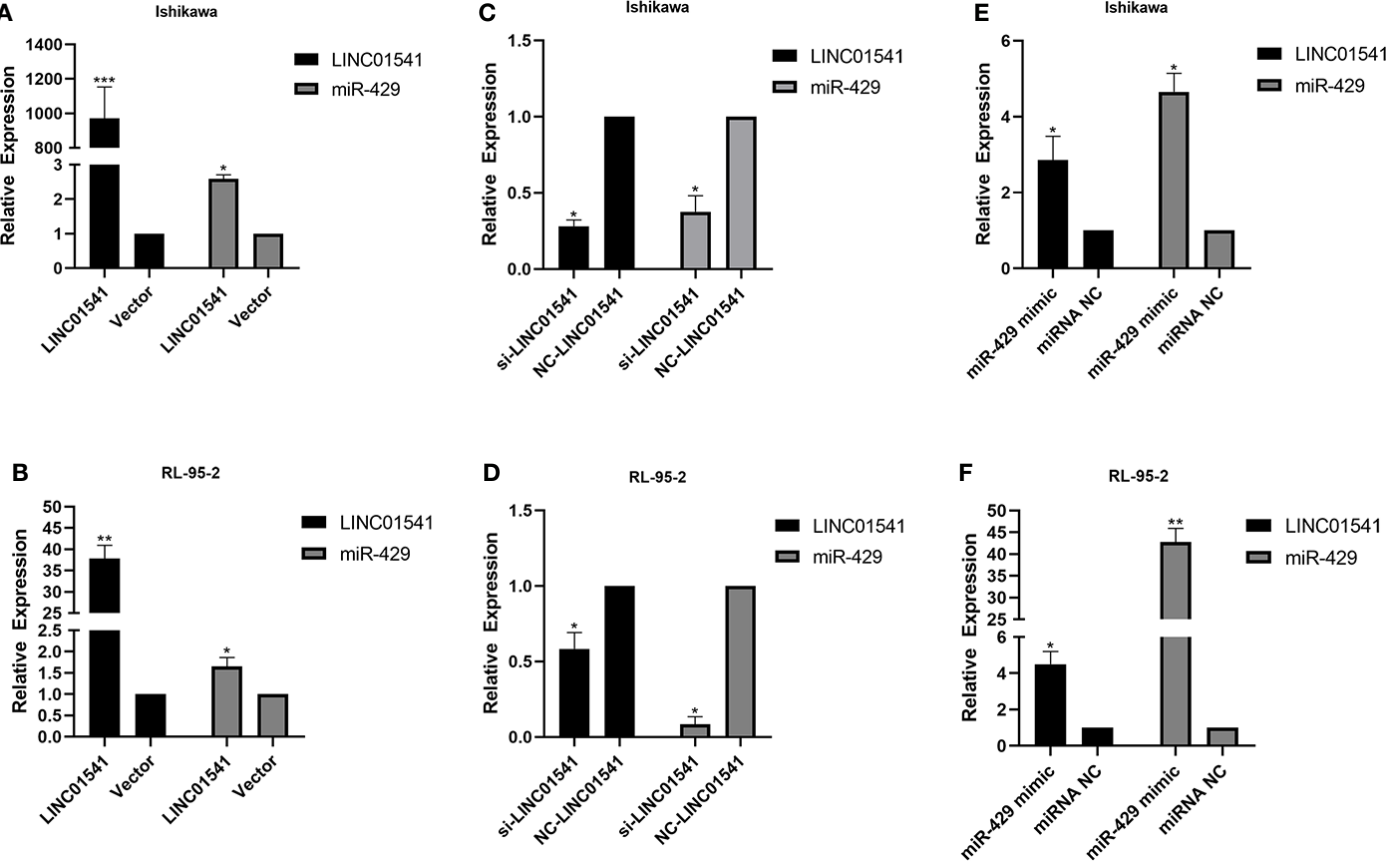
LINC01541 and miR-429 were transfected to measure expression. **(A, B)** After forced overexpression of LINC01541, the expression of LINC01541 and miR-429 were up-regulated in Ishikawa and RL95-2 cells. **(C, D)** When cells were transfected with LINC01541 siRNA, the expression of LINC01541 and miR-429 was down-regulated. **(E, F)** After both cells were treated with miR-429 mimics, the expression of LINC01541 and miR-429 was up-regulated. (*P<0.05; **P<0.01; ***P<0.001).

**Figure 5:**
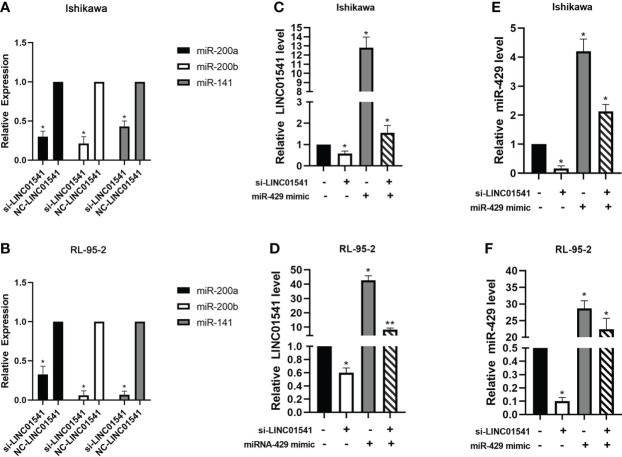
QRT-PCR was used to detect the expression of genes after LINC01541/miR-429 treatment. **(A, B)** Knockdown of LINC01541 was found to inhibit the expression of miR-200a, miR-200b, and miR-141 in Ishikawa and RL95-2 cells. **(C–F)** A co-culture system was constructed with LINC01541 siRNA and miR-429 mimic, and qRT-PCR was used to further verify the relationship between LINC01541 and miR-429 in Ishikawa and RL95-2 cells. (*P<0.05; **P<0.01).

**Figure 6:**
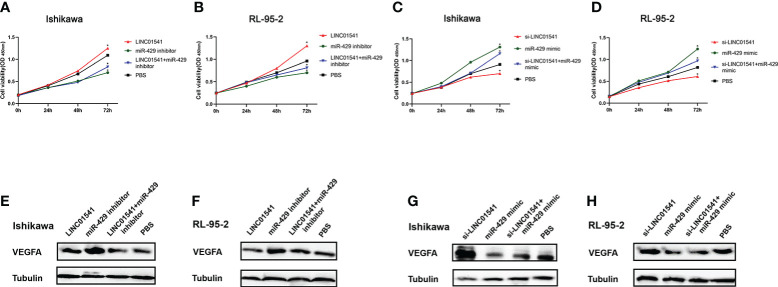
Construction of a co-cultivation system after cell transfections. **(A, B)** CCK-8 used for rescue experiments in Ishikawa and RL95-2 cells demonstrated that LINC01541 promoted cell proliferation, but a mixture of LINC01541 overexpression and miR-429 inhibitor reversed this effect. **(C, D)** In both cell lines, the knockdown of LINC01541 inhibited cell proliferation, but a mixture of LINC01541 knockdown and miR-429 mimic reversed this effect. **(E–H)** Western blot analysis demonstrated that LINC01541 and miR-429 inhibited the production of VEGFA. Western blot analysis used for rescue experiments demonstrated that a mixture of LINC01541 overexpression and miR-429 inhibitor had an intermediate effect on both cell lines. Similarly, a mixture of a knockdown of LINC01541 and miR-429 mimic had an intermediate effect on both cell lines. (*P<0.05).

qRT-PCR demonstrated that LINC01541 and miR-429 interacted to promote each other’s expression (**Figure 4**). The expression of miR-200a, miR-200b, and miR-141 was down-regulated after LINC01541 interference treatment in Ishikawa (**Figure 5A**) and RL95-2 (**Figure 5B**) cells. A mixture of knockdown of LINC01541/miR-429 mimic had an intermediate effect in Ishikawa and RL95-2 cells (**Figures 5C–F**). In addition, LINC01541 was found to promote cell proliferation, but a mixture of LINC01541 overexpression and miR-429 inhibitor was able to reverse this effect in Ishikawa cells (**Figure 6A**) and RL95-2 cells (**Figure 6B**). Similarly, the knockdown of LINC01541 inhibited cell proliferation, but a mixture of LINC01541 knockdown and miR-429 mimic was able to reverse this effect in Ishikawa cells (**Figure 6C**) and RL95-2 cells (**Figure 6D**).

Western blot analysis demonstrated that LINC01541 overexpression and miR-429 mimic inhibited the production of VEGFA, whereas knockdown of LINC01541 and miR-429 inhibitor promoted the production of VEGFA (**Figures 6E–H**). A mixture of LINC01541 overexpression and miR-429 inhibitor, however, had an intermediate effect on Ishikawa cells (**Figure 6E**) and RL95-2 cells (**Figure 6F**). A mixture of knockdown of LINC01541 and miR-429 mimic also had an intermediate effect on Ishikawa (**Figure 6G**) and RL95-2 (**Figure 6H**) cells.

### Bioinformatics analysis and dual luciferase reporter gene detection suggest that miR-429 targets VEGFA

**Figure 7:**
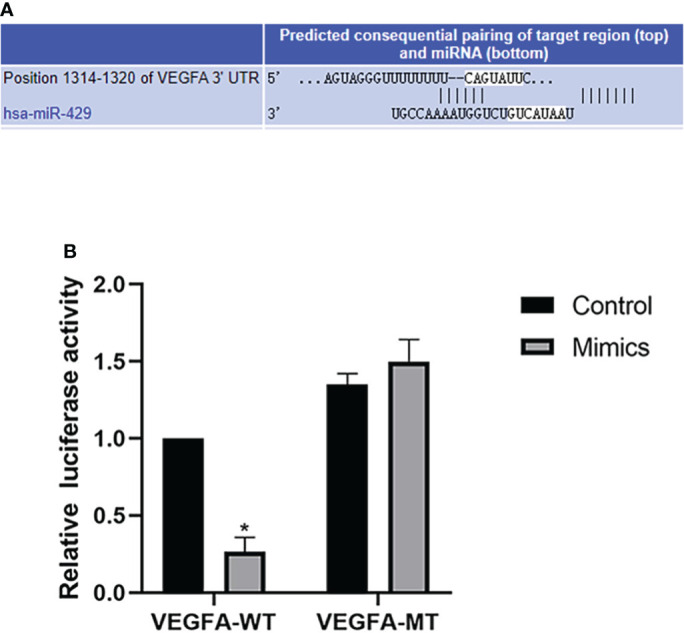
Bioinformatics analysis and dual luciferase reporter gene. **(A)** The binding site of VEGFA and miR-429 was identified with starBase v3.0. **(B)** Dual-luciferase assay results for RL95-2 cells transfected with wild-type (WT) or mutated (Mut) VEGFA reporters plus miR-429 mimic or mimic-NC molecules for 48 h. *P<0.05.

starBase v3.0 software was used to identify the binding site between miR-429 and VEGFA (**Figure 7A**). miR-429 mimic was found to inhibit the production of VEGFA, whereas miR-429 inhibitor promoted the production of VEGFA. Dual-luciferase assay demonstrated that RL95-2 cells cotransfected with miR-429 mimic and VEGFA-WT had less luciferase activity than other groups (**Figure 7B**). These results suggest that VEGFA can directly interact with miR-429.

## Discussion

In this study, we demonstrated that the expression levels of ESR2 and VEGFA were decreased in EAC tissue, whereas the expression levels of LINC01541 miR-200s (except miR-200c) were increased. The GEPIA software predicted that VEGFA would be down-regulated in EAC tissue, which was consistent with our finding that VEGFA expression was decreased in G1/G2 EAC specimens. We also found that E2 treatment of EAC cells inhibited the expression of LINC01541 and miR-429, whereas PHTPP treatment had the opposite effect. E2 treatment of EAC cells was found to inhibit the expression of VEGFA. LINC01541 and miR-429 were found to inhibit the expression of VEGFA and promote the proliferation of EAC cells. In addition, the interaction of LINC01541 and miR-429 promoted the expression of both factors. Finally, through the double validation of bioinformatics analysis and dual fluorescein reporter gene, it was confirmed that miR-429 targets the regulation of VEGFA expression. (* *P* < 0.05; ** *P* < 0.01)

EC is the most frequent gynecological cancer in developed countries and its incidence is increasing, the majority of EC cases are estrogen dependent but the mechanisms of estrogen are not completely understood (2). Previous studies have shown that the mRNA and protein expression levels of ESR2 are significantly lower in more than 100 EC specimens than those in normal endometrium (18–20), and we similarly observed decreased expression of ESR2 in EC samples in our analysis. Hu et al. found that knockdown of E2 in Ishikawa cells promoted cell proliferation (21). *In vivo* study has shown that ER including ESR1, ESR2 and G protein-coupled estrogen receptor (GPER) is essential for a normal menstrual cycle. Incorrect expression of ERs can cause EC, ESR1 promotes uterine cell proliferation and increased the risk of EC, while ESR2 has the opposite effects (22). Other research has shown that LINC01541 inhibits VEGFA expression in endometrial stromal cells and that E2 stimulation significantly inhibits the expression of LINC01541, whereas overexpression of LINC01541 attenuates the migration and invasion of endometrial stromal cells induced by E2 (8). Our findings also support these results. Hormonal therapy is mainly prescribed for fertility preservation with early EC and palliative treatment in patients with advanced or recurrent EC. Studies show that ER pathway activity tests suggest that an active ER pathway may increase sensitivity to EC hormone therapy. High ER pathway activity score (ERPAS) was significantly positively associated with good prognosis and sensitivity to endocrine therapy in EC (23–25).

It is well known that lncRNA can exert its biological function by regulating the expression of miRNA. One group of researchers proposed that lncRNA MT1DP directly binds and stabilizes miR-365 while promoting the expression of miR-365. They found that down-regulation of MT1DP had no effect on the expression of pri-miR-365, suggesting that MT1DP did not regulate miR-365 at the transcriptional level (26, 27). These findings imply that long non-coding RNAs can directly bind and stabilize miRNAs, while promoting the expression of miRNAs, which are consistent with our results. The miR-200 family (miR-200a, miR-200b, miR200c, miR-429, and miR-141) has previously been shown to be up-regulated in EC (28–30). Cui et al. detected 33 EC tissues and 14 endometrial tissue samples and found that miR-141-3p played a positive role in promoting EC cell proliferation (31). Our study did not demonstrate any effect of LINC01541 on pri-miR-429. The specific relationship between LINC01541 and miR-429 needs further study. However, the research results of some scholars have some enlightenment for us. RNA methylation, similar to DNA or protein modification, is regulated by a variety of regulators, including methyltransferases (‘writers’), RNA binding proteins (‘readers’), and demethylases (‘erasers’). Methylation of N6 adenosine (m6A) is the most common type of RNA modification. Alarcón et al. found that methyltransferase-like 3 (METTL3) methylates pri-miRNAs, and *in vitro* experiments also confirmed the role of m6A markers in promoting pri-miRNA processing. In conclusion, alterations in METTL3 expression may significantly affect miRNA expression in various human cancers (32–34). In addition, m6A-lncRNAs have been proven to be involving in regulating tumorigenesis and m6A is likely to participate in the construction of the lncRNA-miRNA-mRNA (ceRNA) interaction regulatory network (35–37). We hypothesized that the interaction between LINC01541 and miR-429 assessed in our study may therefore be m6A-dependent.

The regulatory relationship between miR-429 and VEGFA has been demonstrated in a variety of cancers and systemic diseases. MiR-429 mimics, on the other hand, have been found to reduce the expression of VEGF in clear cell renal cell carcinoma cells (38). Chan et al. proposed that VEGFA with high expression levels was negatively correlated with hsa-miR-429, and inhibition of hsa-miR-429 expression stabilized VEGFA expression in urothelial carcinoma (UC) (39). Ye et al. used bioinformatics analysis software and GEO database to propose miRNA-mRNA pathways such as hsa-miR-429-VEGFA as potential biomarkers for diagnosis and treatment of ovarian cancer (OC) patients (40). Previous research in human umbilical vein endothelial cells has shown that miR-429 can directly bind to the 3’UTR of VEGFA mRNA to directly regulate its expression (41). In cases of neonatal necrotizing enterocolitis, miR-429/200a/200b and miR-141/200c clusters were found to negatively regulate VEGFA, increasing its expression (14). MiR-429 has been shown to increase the expression of VEGF by promoting the synthesis of hypoxia-inducible factor 1α (HIF-1α) in human amniotic mesenchymal stem cells (15).

Gu et al. found that blocking ESR1 or ESR2 can reduce the expression of VEGF and VEGFR in UECC. However, exposure to estrogen accelerated growth and VEGF production in Ishikawa-xenografted nude mice (42). Zhang et al. demonstrated that estrogen-induced angiogenesis appears to occur through induction of various angiogenic factors, such as VEGF and basic fibroblast growth factor (bFGF). Estradiol upregulated mRNA expression and induced protein synthesis of VEGF and bFGF. However, the expression of VEGF and bFGF was blocked by ER inhibitor in estrogen receptor-positive Ishikawa cells (43). One study showed that exogenous therapy with E2 rescues pre-existing advanced heart failure (HF) in mice mainly by regulating ESR2 expression. ESR2 agonists are associated with reduced cardiac fibrosis and increased cardiac angiogenesis (44). In another study, E2 treatment of endometrial stromal cells was demonstrated to be more angiogenic and associated with increased VEGFB protein expression. Meanwhile, these stimulants can be were partially eliminated by an estrogen-receptor antagonist (45). In the current study, miR-200s (except miR-200c) were shown to interact with LINC01541. Because EAC is an estrogen-dependent disease, it is possible that an E2/LINC01541/miR-429/VEGFA axis exists in EAC tissues and that this axis may be a potential therapeutic target.

This study had several limitations. First, the number of samples was also small. Only G1/G2 EAC specimens were collected, and G3 EAC specimens were not included in this study. Secondly, we only conducted CCK-8 experiments to study the effects of 17β-estradiol, estrogen antagonists, LINC01541 and miR-429 on the proliferation of EAC cell lines, and we can also add Edu experiments and plate cloning experiments. Third, we did not study the effect of related genes on the migration, invasion ability and apoptosis of the two cell lines; Fourth, our current work has not examined the effect of LINC01541 on pri-miR-429, and the specific mechanism of the interaction between LINC01541 and miR-429 needs further study; Finally, we have not conducted *in vivo* experiments, and the findings represent only one phenomenon.

## Conclusion

In summary, our results demonstrated that E2 promote the expression of VEGFA by altering LINC01541 and miR-429 expression levels in EAC. We also found that E2-mediated LINC01541/miR-429 expression may affect cell proliferation in EAC. In addition, we identified a reciprocal promotion between LINC01541 and miR-429. These initial results allow us to understand the angiogenesis of EAC from a new perspective and also suggest that the E2-mediated LINC01541/miR-429/VEGFA axis may serve as a new therapeutic target for EAC. More research is needed to validate the diagnostic potential of these genes in larger studies.

## Data availability statement

The original contributions presented in the study are included in the article/supplementary material. Further inquiries can be directed to the corresponding authors.

## Ethics statement

The studies involving human participants were reviewed and approved by The Ethics Committee of Changzhou No. 2 People’s Hospital. The patients/participants provided their written informed consent to participate in this study.

## Author contributions

Conceptualization, ZJ. methodology, SL and ZJ. software, XQ and HY. validation, DQ and XQ. Formal analysis, DQ, HY and XQ. Investigation, LL and XL. Resources, SL. Writing—original draft preparation, DQ. Writing—review and editing, SL and ZJ. All authors have read and agreed to the final version of the manuscript.

## Funding

This study was supported by the hospital level project fund of Changzhou No. 2 People’s Hospital (2020K008). The funder had no role in study design, data collection and analysis, decision to publish, or preparation of the manuscript.

## Conflict of interest

The authors declare that the research was conducted in the absence of any commercial or financial relationships that could be construed as a potential conflict of interest.

The reviewer XQ declared a shared affiliation with the authors XL, LL, SL and ZJ to the handling editor at the time of review.

## Publisher’s note

All claims expressed in this article are solely those of the authors and do not necessarily represent those of their affiliated organizations, or those of the publisher, the editors and the reviewers. Any product that may be evaluated in this article, or claim that may be made by its manufacturer, is not guaranteed or endorsed by the publisher.
